# Astrocytic Toll-Like Receptor 3 Is Associated with Ischemic Preconditioning- Induced Protection against Brain Ischemia in Rodents

**DOI:** 10.1371/journal.pone.0099526

**Published:** 2014-06-10

**Authors:** Lin-na Pan, Wei Zhu, Yang Li, Xu-lin Xu, Lian-jun Guo, Qing Lu, Jian Wang

**Affiliations:** 1 Department of Pharmacology, Tongji Medical College, Huazhong University of Science and Technology, Wuhan, Hubei, People's Republic of China; 2 Medical Department of Neurology, The Second Hospital of Nanchang, Nanchang, Jiangxi, People's Republic of China; 3 Department of Emergency Internal Medicine, Tongji Hospital, Tongji Medical College, Huazhong University of Science and Technology, Wuhan, Hubei, People's Republic of China; 4 Department of Anesthesiology and Critical Care Medicine, Johns Hopkins University, School of Medicine, Baltimore, Maryland, United States of America; 5 The Key Laboratory of Drug Target Research and Pharmacodynamic Evaluation, Hubei Province, Wuhan, Hubei, People's Republic of China; Indian Institute of Integrative Medicine, India

## Abstract

**Background:**

Cerebral ischemic preconditioning (IPC) protects brain against ischemic injury. Activation of Toll-like receptor 3 (TLR3) signaling can induce neuroprotective mediators, but whether astrocytic TLR3 signaling is involved in IPC-induced ischemic tolerance is not known.

**Methods:**

IPC was modeled in mice with three brief episodes of bilateral carotid occlusion. *In vitro*, IPC was modeled in astrocytes by 1-h oxygen-glucose deprivation (OGD). Injury and components of the TLR3 signaling pathway were measured after a subsequent protracted ischemic event. A neutralizing antibody against TLR3 was used to evaluate the role of TLR3 signaling in ischemic tolerance.

**Results:**

IPC *in vivo* reduced brain damage from permanent middle cerebral artery occlusion in mice and increased expression of TLR3 in cortical astrocytes. IPC also reduced damage in isolated astrocytes after 12-h OGD. In astrocytes, IPC or 12-h OGD alone increased TLR3 expression, and 12-h OGD alone increased expression of phosphorylated NFκB (pNFκB). However, IPC or 12-h OGD alone did not alter the expression of Toll/interleukin receptor domain-containing adaptor-inducing IFNβ (TRIF) or phosphorylated interferon regulatory factor 3 (pIRF3). Exposure to IPC before OGD increased TRIF and pIRF3 expression but decreased pNFκB expression. Analysis of cytokines showed that 12-h OGD alone increased IFNβ and IL-6 secretion; 12-h OGD preceded by IPC further increased IFNβ secretion but decreased IL-6 secretion. Preconditioning with TLR3 ligand Poly I:C increased pIRF3 expression and protected astrocytes against ischemic injury; however, cells treated with a neutralizing antibody against TLR3 lacked the IPC- and Poly I:C-induced ischemic protection and augmentation of IFNβ.

**Conclusions:**

The results suggest that IPC-induced ischemic tolerance is mediated by astrocytic TLR3 signaling. This reprogramming of TLR3 signaling by IPC in astrocytes may play an important role in suppression of the post-ischemic inflammatory response and thereby protect against ischemic damage. The mechanism may be via activation of the TLR3/TRIF/IRF3 signaling pathway.

## Introduction

Cerebral ischemic preconditioning (IPC) refers to a transient, sublethal ischemic event that results in tolerance to subsequent lethal cerebral ischemia. IPC is believed to trigger an intrinsic neuroprotective mechanism [1], [2]. Most studies of brain ischemic preconditioning *in vivo* and *in vitro* have been limited to neurons. However, astrocytes comprise the majority of brain cells in mammals and play an important role in the brain's repair and inflammatory responses by producing various cytokines and growth factors [3], [4], [5]. They are essential to preserving neural tissue and restricting inflammation after brain injury. Neurons cannot survive in the brain if adjacent astrocytes are damaged during ischemia or other brain insults [6], [7]. Nonetheless, it remains unknown whether IPC affects astrocyte cell death outcomes after simulated ischemia.

Toll-like receptors (TLRs) play a critical role in initiating the inflammatory response during cerebral injury [8], [9], [10], [11]. TLRs are a family of evolutionarily conserved molecules that directly detect and defend against pathogen invasion. Upon activation by specific ligands, TLR signaling is initiated by two cytoplasmic adaptor proteins: myeloid differentiation factor 88 (MyD88) and Toll/interleukin receptor domain-containing adaptor-inducing IFNβ (TRIF) [12]. Each of the 10 currently known TLR family members, with the exception of TLR3, signals through the MyD88-dependent pathway, which activates transcription factors NF-κB and AP-1 and leads to generation of pro-inflammatory cytokines IL-6 and TNF-α. TLR3 signals through the MyD88-independent pathway (initiated by the adaptor protein TRIF) that activates transcription factors IRF3 and IRF7 and generates anti-inflammatory molecules such as IFNβ and IL-10, which have been associated with neuroprotection [13], [14], [15]. Of the TLRs, only TLR4 can utilize either of these pathways [12].

TLR4 activation in response to cerebral ischemia leads to an excessive inflammatory response that plays a deleterious role in cerebral ischemic injury [16], [17], [18], [19]. However, evidence suggests that TLR4 might also be involved in IPC-induced ischemic tolerance [12], [20], [21]. Studies suggest that preconditioning with TLR4 ligand lipopolysaccharide enhances TLR4 signaling through the MyD88-independent pathway, thereby suppressing the ischemia-induced inflammatory response [22]. Unlike TLR4, TLR3 signals exclusively through the MyD88-independent pathway. Interestingly, deletion of TLR3 in mice did not alter infarction volume after stroke compared with that in wild-type mice [18]. Additionally, Bsibsi et al. reported that medium from human astrocytes conditioned with TLR3 ligand polyinosinic:polycytidylic acid (Poly I:C) improved neuronal survival in human brain slice cultures [23] and that Poly I:C freshly added to control medium promoted neuronal survival equally well. It was also reported that acute treatment of primary mouse cortical cells with Poly I:C protects against oxygen-glucose deprivation (OGD)-induced cell death [24]. Moreover, Poly I:C induces protracted resistance of human astrocytes to H_2_O_2_ toxicity [25], whereas TLR3 contributes to ischemic injury in the gut [26].

In the central nervous system (CNS), astrocytes express robust TLR-3. When activated by cytokines, TLR agonists, or oxidative stress, astrocytes produce TLR3 more strongly than any other TLR [23], [27]. To determine whether astrocytic TLR3 signaling contributes to IPC-induced ischemic tolerance, we established *in vivo* and *in vitro* models of IPC and analyzed expression and function of TLR3 in astrocytes. Furthermore, we examined the potential neuroprotection by preconditioning with TLR3 ligand Poly I:C.

## Materials and Methods

### Animals

All animal protocols were approved by the Institutional Animal Care and Use Committee at the Huazhong University of Science and Technology and conformed to the National Institutes of Health Guide for the Care and Use of Laboratory Animals. One-day-old Sprague-Dawley rats and adult male Kunming mice were obtained from the Center for Experimental Animals, Huazhong University of Science and Technology, China. Thirty newborn rat pups were decapitated after being anesthetized by ether inhalation. We removed the cortex from each rat for astrocyte cultures under sterile conditions. Cerebral ischemia was induced in 24 adult male mice that were anesthetized by intraperitoneal injections of ketamine (100 mg/kg). The mice were placed on a heating pad during surgery to maintain a normal body temperature of 37°C. For the recovery after surgery, the animals were returned to cages with highly absorbent soft bedding. The mice were housed in pairs, and the ambient temperature was maintained at 20–23°C.

### Induction of preconditioning and permanent focal ischemia

During the procedures to induce IPC and focal ischemia, mice were placed under anesthesia with ketamine (100 mg/kg) administered intraperitoneally. For IPC, both common carotid arteries were exposed and ligated with 6-0 silk sutures three times for 1 min each. Between each ligation, the arteries were reopened for 5 min [28]. Sham-operated mice underwent the same procedure without ligation. Permanent focal ischemia was produced by intraluminal middle cerebral artery (MCA) occlusion (MCAO) for 24 h with a 6-0 nylon monofilament (Ethicon, Somerville, NJ, USA). Successful occlusion of the MCA was verified and recorded by laser-Doppler flowmetry (Moor VMS-LDF, Devon, UK). Groups of 9 mice underwent MCAO 24 h after preconditioning or the sham operation.

### Evaluation of neurologic deficit score and determination of infarct size

An investigator blinded to treatment group evaluated the neurologic deficits of each mouse (n = 6 per group) at 24 h after MCAO using the Zea-Longa method as described previously [29], [30]. Mice were then killed with an overdose of pentobarbital. The brains were sectioned into five 1-mm-thick coronal slices and incubated in 2% 2,3,5-triphenyltetrazolium chloride monohydrate (TTC) at 37°C for 15 min, followed by 4% paraformaldehyde overnight. The brain slices were photographed and the area of ischemic damage was measured by an image analysis system [31] (Image J software, a public domain image analysis program developed at the National Institutes of Health).

### Immunofluorescence staining

Immunofluorescence was carried out as described previously [32], [33]. The cellular localization of TLR3 was measured in brain sections from a cohort of mice (n = 3 per group) killed at 24 h after preconditioning; sham-operated mice served as controls. The mice were perfused transcardially with saline followed by 4% paraformaldehyde. The brain region –1.0 mm from the optic chiasm was then cut into 30-µm coronal sections by a cryostat (SM2000R, LEICA, Berlin, Germany). For double staining of TLR3 and glial fibrillary acidic protein (GFAP), the sections were incubated together with goat anti-TLR3 antibody (1∶50; Santa Cruz Biotechnology, Santa Cruz, CA, USA) and mouse anti-GFAP antibody (1∶200; Epitomics, Cambridge, UK) overnight at 4–8°C, and then incubated sequentially with fluorescein-labeled secondary antibody for 2 h at room temperature. Finally, images in the brain cortex were observed with a fluorescence microscope (Olympus BX-51; Olympus Optical, Tokyo, Japan).

### Astrocyte culture

Astrocyte cultures were prepared from neonatal rat cortical cultures as described previously [34]. Briefly, mixed cortical neurons and glia were cultured in 75-cm^2^ flasks at a concentration of 2×10^6^ cells/mL in DMEM/F-12 containing 20% fetal bovine serum, 100 U/mL penicillin, and 100 mg/mL streptomycin. On day 14 of culture, flasks were shaken at 200 rpm for 5 h to detach microglia and oligodendrocytes from the layer of astrocytes, which are more adherent. Astrocytes remaining in the flask were harvested with 0.125% trypsin, and the suspension was centrifuged at 1000 rpm for 10 min. The pellet was resuspended and cultured in flasks at a concentration of 2×10^6^ cells/mL. On day 19 of culture, the flasks were shaken again to exclude microglial contamination [35], and astrocytes remaining in the flasks were harvested. The pellet was resuspended to a concentration of 1–2×10^5^ cells/mL with culture medium containing 20% fetal bovine serum. Cells were plated to achieve a confluent monolayer on 96-well culture plates or 35-mm dishes coated with poly-D-lysine (100 µg/mL). Anti-GFAP antibodies were used to tag microfibers in the cytoplasm of astrocytes. To identify astrocytes, we analyzed cultures by immunofluorescent staining with goat anti-GFAP antibody (Sigma-Aldrich, St. Louis, MO, USA) and counterstained with DAPI to stain nuclei. All experiments were performed on day 22 of culture.

### Oxygen-glucose deprivation (OGD)

The cultures were incubated in glucose-free DMEM in an airtight box that was continuously filled with 95% N_2_ and 5% CO_2_ to induce OGD as described by Liu et al. [36]. Cultures subjected to transient 1-h OGD and then reoxygenated for 24 h to induce OGD resistance were designated as the IPC group based on our trial experiment. Cultures subjected to 12-h OGD (lethal) were designated as the OGD group. Cultures that were exposed to 1-h OGD 1 day before being subjected to 12-h OGD were designated as the IPC+OGD group. To evaluate the role of TLR3 signaling in simulated ischemic injury, we treated a portion of the astrocytes with 50 ng/mL neutralizing antibody against TLR3 (Ab-TLR3, Santa Cruz Biotechnology) or rabbit non-immune IgG 2 h before OGD. We also evaluated the protective effect of TLR3 ligand Poly I:C in astrocytes. The cells were treated with 5 or 10 µg/mL Poly I:C or Poly I:C plus 50 ng/mL Ab-TLR3 24 h before being subjected to 12-h OGD. Cultures exposed to normoxia served as controls.

### Cell viability assay

The ability of cells to convert MTT into formazan is an indication of mitochondrial integrity and activity, which acts as a surrogate for cell viability [37]. Briefly, MTT was added to cells at a final concentration of 0.5 mg/mL. After 4-h incubation at 37°C, the blue reaction product, formazan, was dissolved by 100 µL of DMSO. The absorbance value at 570 nm was determined with a microplate reader. Results are expressed as percentages of control values. Cell viability was measured in triplicate for each experimental condition.

### Lactate dehydrogenase (LDH) release assay

Cytotoxicity after OGD was determined by measuring LDH released into the culture medium with a Cytotoxicity Detection Kit (Roche, Basel, Switzerland) as described previously [38]. Absorbance at 492 nm was determined on a microplate reader. LDH values were normalized to the mean maximal LDH value in sister cultures continuously exposed to 0.1% Triton X-100, which produces near-complete glial cell death (100%) [39]. LDH release was measured in triplicate for each experimental condition.

### Protein extraction and Western blot analysis for TLR3, TRIF, phosphorylated IRF3 (pIRF3), and phosphorylated-NFκB p65 (p-NFκB p65)

Astrocyte cultures were washed with ice-cold phosphate-buffered saline (PBS), and the proteins were extracted with 80 µL of lysis buffer. For pIRF3 and p-NFκB p65 protein extraction, the lysis buffer contained a mix of protease and phosphorylated protease inhibitors. Protein concentration in each sample was determined according to the Bradford method, with serum albumin as a standard. Equal amounts of the protein samples were loaded onto 12% sodium dodecyl sulfate–polyacrylamide gels, separated by electrophoresis, and transferred onto a polyvinylidene difluoride membrane. Membranes were incubated with rabbit anti-TLR3 polyclonal antibody (1∶1000; Abcam, Cambridge, UK), rabbit anti-TRIF polyclonal antibody (1∶600; Abcam), rabbit anti-pIRF3 (1∶500; Anbo, San Francisco, CA, USA), rabbit anti-p-NFκB p65 polyclonal antibody (1∶500; Santa Cruz Biotechnology), or goat anti-actin (1∶500; Santa Cruz Biotechnology) at 4°C overnight. Membranes were then incubated with horseradish peroxidase-conjugated secondary antibodies for 1 h at room temperature. The positive bands were revealed with enhanced chemiluminescence detection reagents and autoradiography film. Optical densities of the bands were scanned and quantified with Image J software. Actin served as an internal control.

### Immunofluorescence staining of TLR3

For immunofluorescence staining, cells on coverslips were fixed in 4% paraformaldehyde for 10 min, permeabilized with 0.1% Triton X-100 for 60 min, and then incubated with rabbit anti-TLR3 polyclonal antibody (1∶200; Abcam) for 2 h at room temperature. After being washed thoroughly, the cells were incubated with FITC-conjugated anti-rabbit antibody for 1.5 h at room temperature. The coverslips were washed again and mounted onto glass microscope slides with mounting medium. Cell staining was viewed under the fluorescence microscope (Olympus BX-51; Olympus Optical, Tokyo, Japan).

### ELISA assay for cytokine release

IFNβ and IL-6 secreted into the culture medium were measured with commercially available ELISA kits (R&D Systems, Minneapolis, MN, USA) according to the manufacturer's instructions. Absorbance at 450 nm was determined on a microplate reader.

### Statistical analysis

All data are expressed as mean ± SD. The statistical analyses were carried out by one-way ANOVA with SPSS for Windows (Version 18.0). Ranked data of neurologic deficit were analyzed by the nonparametric Kruskal-Wallis test. Differences were considered significant at *p*<0.05.

## Results

### IPC attenuates MCAO-induced infarct volume and reduces neurologic deficits in mice

**Figure 1:**
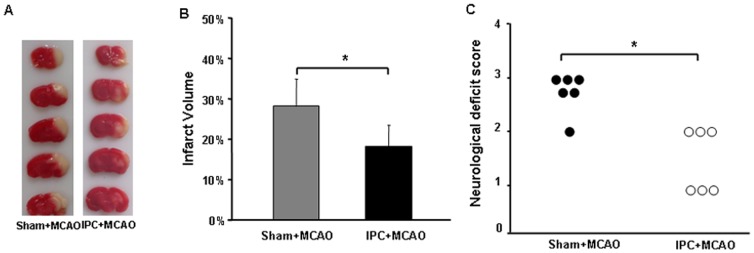
Ischemic preconditioning (IPC) attenuates brain damage caused by middle cerebral artery occlusion (MCAO) in mice. The time interval between preconditioning and ischemia was 24(A) Representative triphenyltetrazolium chloride-stained brain sections (1-mm thick) 24 h after MCAO in sham-operated and preconditioned mice. (B) IPC significantly reduced MCAO-induced Infarct volume compared with that in sham+MCAO controls. (C) IPC significantly attenuated MCAO-induced neurologic deficit compared with that in sham+MCAO controls. Values are expressed as mean ± SD; n = 6 per group; *p<0.05.

IPC alone caused no brain infarct injury and no signs of neurologic deficit (data not shown). Compared with mice that did not undergo IPC, those that underwent IPC before MCAO had smaller infarct volumes in the ipsilateral hemisphere (Figure 1A, B). Mice that received IPC also had lower neurologic deficit scores than did those that did not undergo preconditioning before MCAO (*p*<0.05; Fig.1C).

### Expression of TLR3 after IPC

**Figure 2:**
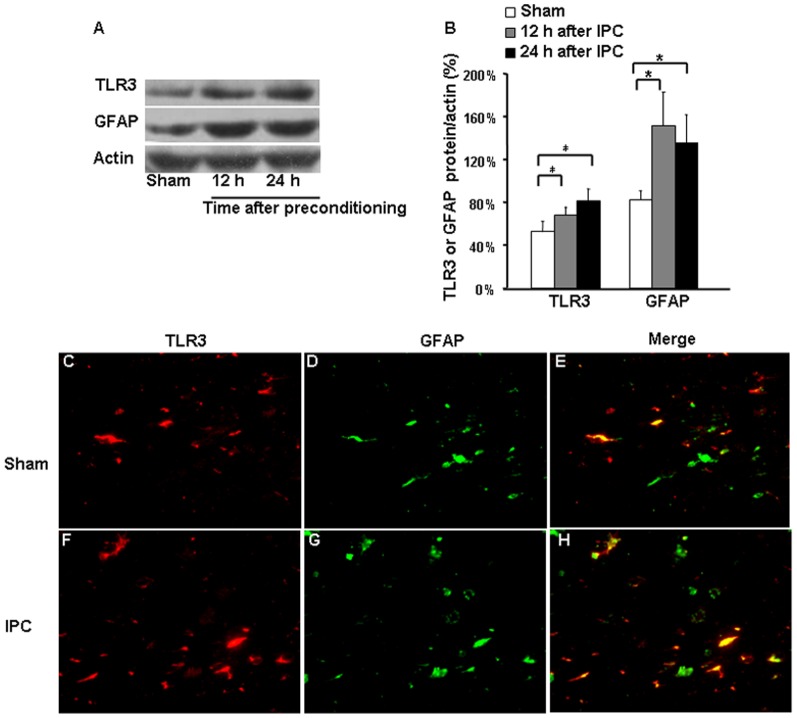
TLR3 and GFAP expression in cerebral cortex after ischemic preconditioning (IPC). (A and B) Western blotting showed that protein expression of Toll-like receptor 3 (TLR3) and glial fibrillary acidic protein (GFAP) was elevated at 12 h and 24 h after IPC compared with that in sham-operated controls. (C and F) TLR3 immunoreactivity. (D and G) GFAP immunoreactivity. (E and H) Merged images show that co-localization of TLR3 and GFAP was increased at 24 h after IPC. Values are expressed as mean ± SD; n = 3 per group; *p<0.05. Magnification in C-H: 200x.

Expression levels of TLR3 and GFAP in preconditioned mice were upregulated at 12 and 24 h after IPC compared with those in sham-operated animals (Figure 2A, B). Additionally, TLR3 immunoreactivity colocalized with GFAP-positive astrocytes (Figure 2C-H). In the brain cortex, TLR3 expression was significantly elevated in GFAP-positive astrocytes at 24 h after IPC compared with that after the sham procedure.

### IPC protects cultured astrocytes against OGD-induced injury

**Figure 3:**
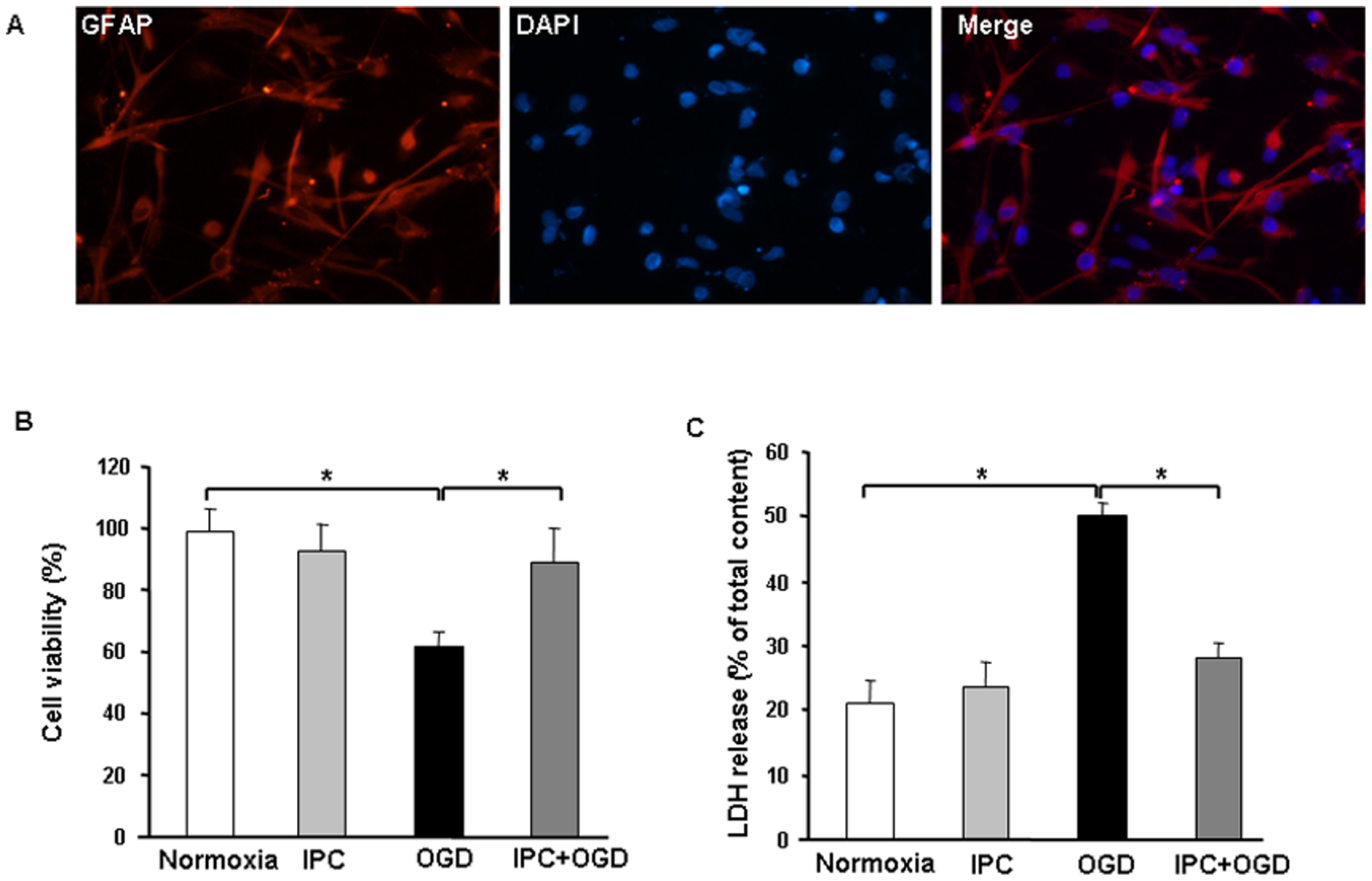
Ischemic preconditioning (IPC) of astrocytes before 12-h oxygen-glucose deprivation (OGD) protects cells against OGD-induced ischemic injury. (A) Astrocytes labeled for GFAP appear bright red, and cell nuclei are stained blue with DAPI. Analysis of the cultures showed that approximately 95% of cells were GFAP-positive. Magnification: 400x. (B and C) IPC before OGD significantly increased cell viability as measured by MTT assay (B) and lactate dehydrogenase (LDH) release (C). Values are expressed as mean ± SD of three independent experiments, each carried out in triplicate; *p<0.05.

Cell cultures were composed of more than 95% astrocytes, as identified by a comparison of GFAP-positive and DAPI-positive cells (Figure 3A). Sublethal 1-h OGD (IPC) followed by 24 h of reperfusion had no effect on cell viability as determined by MTT assay (IPC: 92.4±8.7%, control: 98.4±8.2%; *p*>0.05; Figure 3B). However, exposure of astrocytes to 12-h OGD significantly decreased viability compared with that of the normoxia group (61.6±5.1%, *p*<0.05). Subjecting cultured astrocytes to IPC 24 h before 12-h OGD resulted in a significant increase in cell viability compared with that in the OGD group (89.3±11.3%, *p*<0.05; Figure 3B). LDH release, another indicator of cell toxicity, confirmed the protective effect of IPC. Sublethal 1-h OGD followed by 24 h of reperfusion had no effect on LDH release compared with that in the normoxia group (23.4±4.0% vs. 21.3±3.0%, respectively, *p*>0.05; Figure 3C). In contrast, 12-h OGD significantly increased LDH release to 50.0±2.5% (*p*<0.05 vs. control). Subjecting cultured astrocytes to IPC 24 h before 12-h OGD significantly reduced LDH release compared with that in the OGD group (28.1±2.2%, *p*<0.05). These results suggest that IPC protects astrocytes against OGD-induced cell injury.

### Effects of IPC and OGD on TLR3/TRIF/pIRF3 protein expression in astrocyte cultures

**Figure 4:**
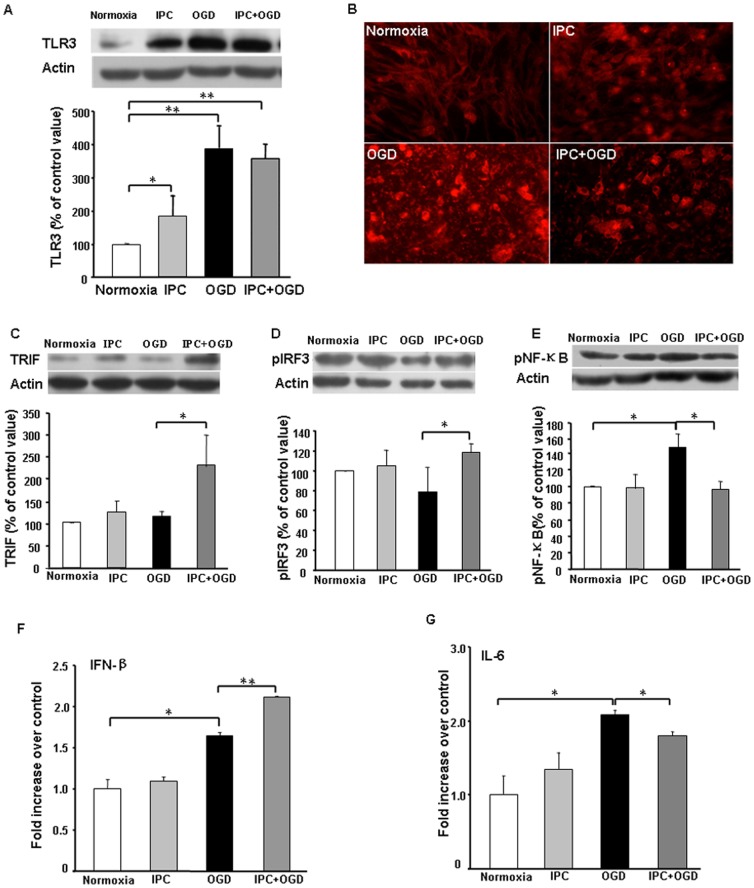
Ischemic preconditioning (IPC) of astrocytes regulates TLR3, TRIF, pIRF3, and NF-κB expression and cytokine release after oxygen-glucose deprivation (OGD). (A and B) Protein expression of TLR3 was upregulated after IPC and OGD, as measured by Western blot analysis (A) and immunofluorescence staining (B). (C and D) IPC of astrocytes before OGD enhanced protein expression of TRIF (C) and pIRF3 (D). (E) IPC prevented the OGD-induced upregulation of pNF-κB protein expression in astrocytes. (F) IPC+OGD enhanced the release of IFNβ compared to levels in the OGD group. (G) IPC+OGD decreased IL-6 release compared to levels in the OGD group. Values are expressed as mean ± SD of three independent experiments, each carried out in triplicate; *p<0.05, **p<0.01.

To investigate the potential involvement of TLR3 in ischemic tolerance, we measured its expression by Western blot and immunofluorescence staining (Figure 4A, B). Sublethal 1-h OGD alone significantly increased TLR3 expression compared with that of the normoxia group. TLR3 expression was elevated even more after 12-h OGD, regardless of preconditioning. Although IPC significantly increased the expression of TLR3, it did not alter the expression of TRIF or pIRF3 compared with that in the normoxia group (Figure 4C, D). Twelve hours of OGD had no effect on TRIF and caused a slight, but non-significant, decrease in pIRF3. However, the IPC+OGD group exhibited significant increases in TRIF and pIRF3 expression compared with that in the OGD-only group (Figure 4C, D). These data indicate that TLR3 expression is induced quickly by IPC and that IPC prior to OGD can promote TRIF and pIRF3 expression during subsequent OGD injury.

### IPC prevents upregulation of p-NFκB p65 protein expression in cultured astrocytes after simulated ischemia

Activation of NFκB protein is associated with pro-inflammatory responses. Using Western blots, we examined p-NFκB p65 protein levels in the different groups (Figure 4E). IPC alone did not alter p-NFκB p65 expression compared with that of the normoxia group, but 12-h OGD significantly increased p-NFκB p65 expression in astrocytes. Exposure of astrocytes to IPC prevented the OGD-induced increase in p-NFκB p65 expression.

### IPC increases IFNβ and attenuates IL-6 release in cultured astrocytes after simulated ischemia

To determine whether IPC regulates the balance of pro- and anti-inflammatory cytokines released by astrocytes *in vitro*, we examined the levels of IFNβ and IL-6 in the culture medium. Compared with levels in the normoxia group, IPC alone did not alter levels of IFNβ and IL-6, whereas 12-h OGD injury caused a significant increase in IFNβ and IL-6 release. However, IPC before OGD further promoted IFNβ release and mitigated OGD-induced IL-6 release (Figure 4F, G).

### Poly I:C-mediated TRIF/IRF3 signaling protects against OGD *in vitro*

Increased levels of TLR3, TRIF, pIRF3, and IFNβ in response to IPC followed by OGD support a possible protective mechanism of astrocytic TLR3 signaling. This signaling may result in a TRIF/pIRF3-mediated anti-inflammatory response. We used TLR3 ligand Poly I:C to determine whether TLR3 activation induces protection.

**Figure 5:**
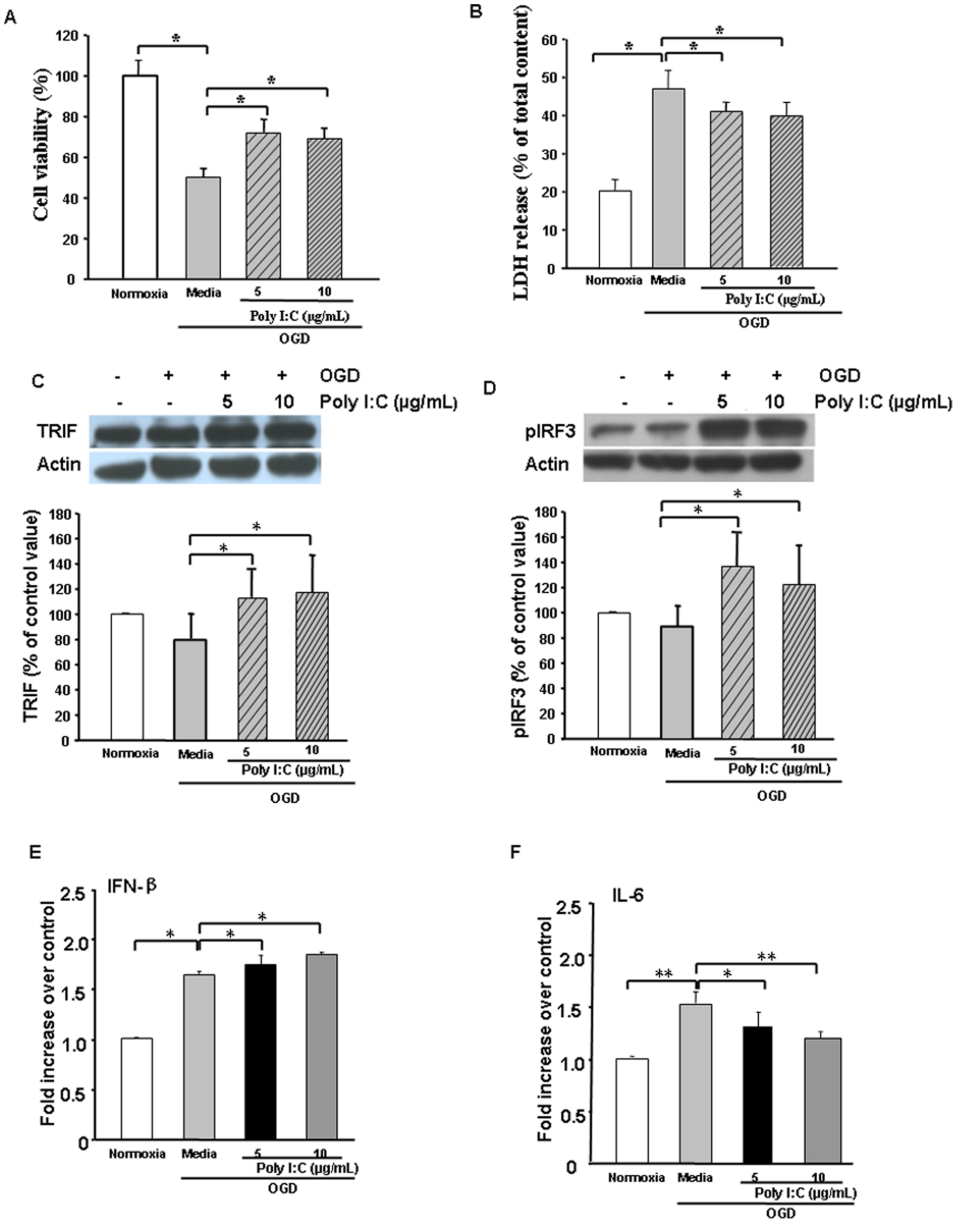
Poly I:C pretreatment reduces oxygen-glucose deprivation (OGD)–induced astrocyte injury. Astrocyte cultures were treated with 5 µg/mL or 10 µg/mL Poly I:C 24 h before OGD. (A and B) Poly I:C pretreatment reduced OGD-induced astrocyte injury as measured by MTT assay (A) and lactate dehydrogenase (LDH) release (B). (C and D) Western blot analysis showed that Poly I:C pretreatment increased the expression of TRIF protein (C) and pIRF3 protein (D) in astrocytes subjected to 12-h OGD. (E) ELISA showed that OGD alone significantly increased IFNβ release and that Poly I:C pretreatment increased IFNβ release further. (F) ELISA showed that OGD alone significantly increased IL-6 release but that Poly I:C pretreatment inhibited the increase in IL-6 release. Values are expressed as mean ± SD of three independent experiments, each carried out in triplicate; *p<0.05, **p<0.01.

At both doses tested, Poly I:C treatment 24 h before OGD significantly reduced OGD-induced astrocyte injury as measured by MTT and LDH assay (Figure 5A, B). Poly I: C pretreatment significantly increased TRIF and pIRF3 expression (Figure 5C, D) and promoted IFNβ release (Figure 5E) but attenuated IL-6 secretion in ischemic astrocytes (Figure 5F). These data suggest that TLR3 signaling may mediate protection in astrocytes.

### TLR3 is required for IPC- or Poly I:C preconditioning-induced protection against simulated ischemia in astrocytes

**Figure 6:**
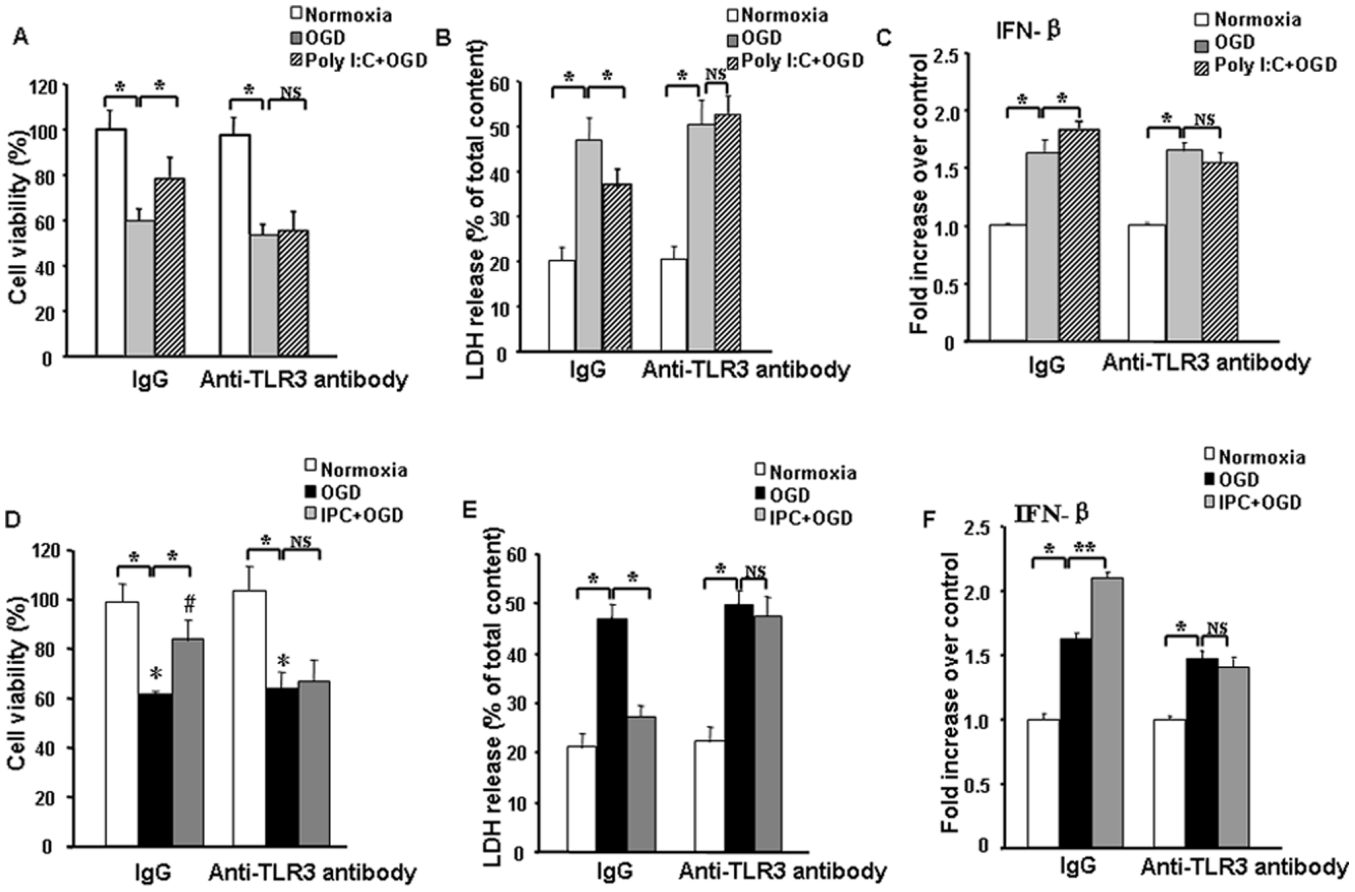
TLR3 signaling is required for ischemic preconditioning (IPC)- or Poly I:C preconditioning-induced protection in astrocytes. (A–C) Astrocyte cultures were treated with 10 µg/mL Poly I:C plus non-immune IgG or 50 ng/mL anti-TLR3 antibody (Ab-TLR3) 24 h before being subjected to 12-h oxygen-glucose deprivation (OGD). (D–F) Astrocyte cultures were exposed to IPC and treated with non-immune IgG or 50 ng/mL Ab-TLR3 24 h before being subjected to 12-h OGD. Pretreatment of astrocyte cultures with the neutralizing antibody against TLR3 reversed IPC- and Poly I:C-induced ischemic protection and the increase in IFNβ. Values are expressed as mean ± SD of three independent experiments, each carried out in triplicate; *p<0.05, **p<0.01, NS  =  not significant; LDH  =  lactate dehydrogenase.

Increased expression of TRIF, pIRF3, and IFNβ after simulated ischemia in IPC- or Poly I:C-preconditioned astrocytes suggests pivotal participation of TLR3 signaling in the preconditioning. To further confirm this hypothesis, we pretreated the preconditioned cells with anti-TLR3 neutralizing antibody. Without preconditioning, cells pretreated with anti-TLR3 neutralizing antibody and nonspecific IgG showed similar degrees of cell injury after OGD, as measured by MTT and LDH assay. Notably, however, blockade of TLR3 signaling with the neutralizing antibody reversed IPC- and Poly I:C-induced ischemic protection and augmentation of IFNβ; use of nonspecific antibody had no effect (Figure 6A–F). These results confirm that TLR3 signaling is involved in IPC- and Poly I:C-induced protection of astrocytes during ischemic conditions.

## Discussion

In our study, we examined the protective potential of IPC *in vivo* and *in vitro* to clarify the role of astrocytes in IPC-induced cerebral ischemia tolerance. Our results showed that IPC *in vivo* with three brief episodes of bilateral carotid artery occlusion reduced brain damage in a permanent focal cerebral ischemia model and that IPC *in vitro* with transient 1-h OGD reduced post-injurious OGD-induced damage to astrocytes. We observed increases in astrocytic TLR3 expression after IPC both *in vivo* and *in vitro*. Astrocyte function is thought to be required for neuronal survival and functional recovery after cerebral ischemia [40]. IPC-induced protection against ischemia may be associated with preserving astrocyte function during the post-ischemic period.

TLR3 is expressed throughout the brain, mostly on astrocytes [12]. It is the only TLR that signals exclusively through the MyD88-independent pathway, which activates TRIF and IRF3 and results in production of anti-inflammatory mediators such as IFNβ, IL-10, TGFβ, and RANTES [41]. Most downstream products of the MyD88-independent pathway have been shown to protect neurons from ischemic damage. For example, direct administration of IFNβ reduced ischemic brain damage in rat and rabbit models of ischemic stroke [13], [14], [42]. IFNβ has already been approved for treatment of multiple sclerosis in humans [43]. The protective effects of IFNβ are associated with reduced neutrophil infiltration and attenuated blood–brain barrier damage [14]. To explore whether IPC-induced neuroprotection is related to astrocytic TLR3 signaling, we examined TLR3, TRIF, and pIRF3 protein expression in cultured ischemic astrocytes, as well as IFNβ levels in the culture medium. We found that transient IPC alone and lethal OGD exposure each dramatically enhanced TLR3 expression in astrocytes, suggesting that TLR3 signaling is activated during IPC and that pre-activation of TLR3 in astrocytes may contribute to neuroprotection induced by IPC. Despite upregulation of TLR3 protein, expression of neither TRIF nor pIRF3 was changed after IPC alone or lethal OGD alone. In contrast, both proteins were increased in the IPC+OGD group, suggesting that transient ischemia primes the pathway for a later upregulation of TRIF and pIRF3 during a lethal ischemic insult. This mobilized adaptation of TLR3 prior to ischemia may activate TRIF and pIRF3 signaling and then increase IFNβ release during subsequent ischemia. Indeed, Marsh et al. [24] reported that mice lacking TRIF/IRF3 were not protected by exogenous lipopolysaccharide preconditioning in an *in vivo* stroke model.

It has been demonstrated that NF-κB activation plays a critical role in the response to cerebral ischemic injury [16], [17]. Activation of NF-κB produces pro-inflammatory factors and aggravates neurologic impairments [8] Thus, inhibition of NF-κB strongly protects against cerebral ischemia. Our results revealed downregulation of TLR4 downstream signaling molecule pNF-κB and decreased levels of IL-6 when IPC preceded 12-h OGD, suggesting that the protective effects of IPC in ischemic astrocytes are also mediated by downregulation of the NF-κB signaling pathway. Relatively high expression of TLR3 may ensure that IPC induces protection in astrocytes by enhancing signaling via the TRIF/IRF3 pathway and thus suppressing signaling via the NF-κB pathway. It has been shown that sublethal preconditioning induces expression of pro-inflammatory cytokines such as IL-1β, TNF-α, and IL-6 [44], which can significantly induce TLR3 expression in astrocytes [23]. In our study, we observed a slight increase in IL-6 after IPC in astrocytes. The release of small amounts of cytokines from cells may partly contribute to TLR3 signal activation during preconditioning and then induce expression of a range of neuroprotective mediators. It has been reported previously that several downstream products of IRF3, such as TRIM30-α, negatively regulate the NF-κB signaling pathway [45]. However, the exact molecular mechanisms by which TRIF and IRF3 mediate downregulation of the NF-κB pathway require further study.

Poly I:C activation of TLR3, which signals through a TRIF-dependent pathway, induces expression of various neuroprotective mediators and anti-inflammatory cytokines in human astrocytes [23]. Borysiewicz et al. [25] reported that TLR3 ligation with Poly I:C up to 2 µg/mL protects astrocytes against oxidative stress. Another study reported that acute Poly I:C treatment up to100 µg/mL significantly reduced OGD–mediated cell death in mixed cortical cultures from mice [24]. We and others have shown that Poly I:C preconditioning provides neuroprotection against cerebral ischemia *in vivo* [46], [47], [48]. Here, we show that Poly I:C also induces ischemic resistance in astrocytes. Preconditioning with 5 or 10 µg/mL Poly I:C significantly reduced OGD-induced cell death and LDH release, increased TRIF and pIRF3 protein expression, enhanced IFNβ release, and decreased IL-6 release. Poly I:C activation of astrocytes triggered a 2.9-fold increase in interferon regulatory factor-1 expression [23], and Poly I:C activation of monocytes triggered a 100-fold increase in IFNβ production [49]. We found that IFNβ increased approximately twofold over the control level after Poly I:C treatment. These discrepancies may be the result of species specificity or differences in sensitivity of detection methods. Because Poly I:C activates not only TLR3 but also at least two other cytosolic receptors, MDA-5 and Rig-I [50], we confirmed involvement of TLR3 signaling in Poly I:C-induced ischemic tolerance by using TLR3 neutralizing antibody. Poly I:C preconditioning-induced protection may be related to activation of TRIF-pIRF3 signaling via TLR3 in astrocytes, which, in turn, would enhance production of anti-inflammatory cytokines in the ischemic astrocytes. Additionally, Gesuete et al. [46] indicated that Poly I:C preconditioning might attenuate blood–brain barrier dysfunction through induction of IFNβ.

IPC in the brain is a natural phenomenon that likely protects against ischemic brain injury by preventing inflammation. Our results indicate that activation of the TLR-TRIF-pIRF3 signaling pathway in astrocytes by IPC or Poly I:C preconditioning could contribute to the mechanism by which the post-ischemic inflammatory response is suppressed. To the best of our knowledge, our study is the first to show that IPC can protect astrocytes against simulated ischemia *in vitro* and that the mechanism may be related to the activation of the TLR3-TRIF-IRF3 signaling pathway.

## References

[1] Shpargel KB, Jalabi W, Jin Y, Dadabayev A, Penn MS, et al (2008) Preconditioning paradigms and pathways in the brain. Cleve Clin J Med 75 Suppl 2 S77–S82. 18540152 10.3949/ccjm.75.suppl_2.s77

[2] Liu XQ, Sheng R, Qin ZH (2009) The neuroprotective mechanism of brain ischemic preconditioning. Acta Pharmacol Sin 30: 1071–1080. 19617892 10.1038/aps.2009.105 PMC4006675

[3] Wang J, Dore S (2007) Inflammation after intracerebral hemorrhage. J Cereb Blood Flow Metab 27: 894–908. 17033693 10.1038/sj.jcbfm.9600403

[4] Wang J (2010) Preclinical and clinical research on inflammation after intracerebral hemorrhage. Prog Neurobiol 92: 463–477. 20713126 10.1016/j.pneurobio.2010.08.001 PMC2991407

[5] Li L, Lundkvist A, Andersson D, Wilhelmsson U, Nagai N, et al (2008) Protective role of reactive astrocytes in brain ischemia. J Cereb Blood Flow Metab 28: 468–481. 17726492 10.1038/sj.jcbfm.9600546

[6] Barreto G, White RE, Ouyang Y, Xu L, Giffard RG (2011) Astrocytes: targets for neuroprotection in stroke. Cent Nerv Syst Agents Med Chem 11: 164–173. 21521168 10.2174/187152411796011303 PMC3167939

[7] Gabryel B, Trzeciak HI (2001) Role of astrocytes in pathogenesis of ischemic brain injury. Neurotox Res 3: 205–221. 14715474 10.1007/BF03033192

[8] Harari OA, Liao JK (2010) NF-kappaB and innate immunity in ischemic stroke. Ann N Y Acad Sci 1207: 32–40. 20955423 10.1111/j.1749-6632.2010.05735.x PMC3807097

[9] Lakhan SE, Kirchgessner A, Hofer M (2009) Inflammatory mechanisms in ischemic stroke: therapeutic approaches. J Transl Med 7: 97. 19919699 10.1186/1479-5876-7-97 PMC2780998

[10] Kilic U, Kilic E, Matter CM, Bassetti CL, Hermann DM (2008) TLR-4 deficiency protects against focal cerebral ischemia and axotomy-induced neurodegeneration. Neurobiol Dis 31: 33–40. 18486483 10.1016/j.nbd.2008.03.002

[11] Zhou Y, Wang Y, Wang J, Anne SR, Yang QW (2014) Inflammation in intracerebral hemorrhage: From mechanisms to clinical translation. Prog Neurobiol 115C: 25–44. 10.1016/j.pneurobio.2013.11.003 24291544

[12] Marsh BJ, Stenzel-Poore MP (2008) Toll-like receptors: novel pharmacological targets for the treatment of neurological diseases. Curr Opin Pharmacol 8: 8–13. 17974478 10.1016/j.coph.2007.09.009 PMC2674015

[13] Veldhuis WB, Derksen JW, Floris S, Van Der Meide PH, De Vries HE, et al (2003) Interferon-beta blocks infiltration of inflammatory cells and reduces infarct volume after ischemic stroke in the rat. J Cereb Blood Flow Metab 23: 1029–1039. 12973019 10.1097/01.WCB.0000080703.47016.B6

[14] Veldhuis WB, Floris S, van der Meide PH, Vos IM, de Vries HE, et al (2003) Interferon-beta prevents cytokine-induced neutrophil infiltration and attenuates blood-brain barrier disruption. J Cereb Blood Flow Metab 23: 1060–1069. 12973022 10.1097/01.WCB.0000080701.47016.24

[15] Grilli M, Barbieri I, Basudev H, Brusa R, Casati C, et al (2000) Interleukin-10 modulates neuronal threshold of vulnerability to ischaemic damage. Eur J Neurosci 12: 2265–2272. 10947805 10.1046/j.1460-9568.2000.00090.x

[16] Cao CX, Yang QW, Lv FL, Cui J, Fu HB, et al (2007) Reduced cerebral ischemia-reperfusion injury in Toll-like receptor 4 deficient mice. Biochem Biophys Res Commun 353: 509–514. 17188246 10.1016/j.bbrc.2006.12.057

[17] Caso JR, Pradillo JM, Hurtado O, Lorenzo P, Moro MA, et al (2007) Toll-like receptor 4 is involved in brain damage and inflammation after experimental stroke. Circulation 115: 1599–1608. 17372179 10.1161/CIRCULATIONAHA.106.603431

[18] Hyakkoku K, Hamanaka J, Tsuruma K, Shimazawa M, Tanaka H, et al (2010) Toll-like receptor 4 (TLR4), but not TLR3 or TLR9, knock-out mice have neuroprotective effects against focal cerebral ischemia. Neuroscience 171: 258–267. 20804821 10.1016/j.neuroscience.2010.08.054

[19] Tang SC, Arumugam TV, Xu X, Cheng A, Mughal MR, et al (2007) Pivotal role for neuronal Toll-like receptors in ischemic brain injury and functional deficits. Proc Natl Acad Sci U S A 104: 13798–13803. 17693552 10.1073/pnas.0702553104 PMC1959462

[20] Pradillo JM, Fernandez-Lopez D, Garcia-Yebenes I, Sobrado M, Hurtado O, et al (2009) Toll-like receptor 4 is involved in neuroprotection afforded by ischemic preconditioning. J Neurochem 109: 287–294. 19200341 10.1111/j.1471-4159.2009.05972.x

[21] Marsh BJ, Williams-Karnesky RL, Stenzel-Poore MP (2009) Toll-like receptor signaling in endogenous neuroprotection and stroke. Neuroscience 158: 1007–1020. 18809468 10.1016/j.neuroscience.2008.07.067 PMC2674023

[22] Vartanian KB, Stevens SL, Marsh BJ, Williams-Karnesky R, Lessov NS, et al (2011) LPS preconditioning redirects TLR signaling following stroke: TRIF-IRF3 plays a seminal role in mediating tolerance to ischemic injury. J Neuroinflammation 8: 140. 21999375 10.1186/1742-2094-8-140 PMC3217906

[23] Bsibsi M, Persoon-Deen C, Verwer RW, Meeuwsen S, Ravid R, et al (2006) Toll-like receptor 3 on adult human astrocytes triggers production of neuroprotective mediators. Glia 53: 688–695. 16482523 10.1002/glia.20328

[24] Marsh B, Stevens SL, Packard AE, Gopalan B, Hunter B, et al (2009) Systemic lipopolysaccharide protects the brain from ischemic injury by reprogramming the response of the brain to stroke: a critical role for IRF3. J Neurosci 29: 9839–9849. 19657036 10.1523/JNEUROSCI.2496-09.2009 PMC2946887

[25] Borysiewicz E, Doppalapudi S, Kirschman LT, Konat GW (2013) TLR3 ligation protects human astrocytes against oxidative stress. J Neuroimmunol 255: 54–59. 23245579 10.1016/j.jneuroim.2012.11.002

[26] Cavassani KA, Ishii M, Wen H, Schaller MA, Lincoln PM, et al (2008) TLR3 is an endogenous sensor of tissue necrosis during acute inflammatory events. J Exp Med 205: 2609–2621. 18838547 10.1084/jem.20081370 PMC2571935

[27] McCusker RH, Kelley KW (2013) Immune-neural connections: how the immune system's response to infectious agents influences behavior. J Exp Biol 216: 84–98. 23225871 10.1242/jeb.073411 PMC3515033

[28] Cho S, Park EM, Zhou P, Frys K, Ross ME, et al (2005) Obligatory role of inducible nitric oxide synthase in ischemic preconditioning. J Cereb Blood Flow Metab 25: 493–501. 15689953 10.1038/sj.jcbfm.9600058

[29] Lu Q, Xia N, Xu H, Guo L, Wenzel P, et al (2011) Betulinic acid protects against cerebral ischemia-reperfusion injury in mice by reducing oxidative and nitrosative stress. Nitric Oxide 24: 132–138. 21292018 10.1016/j.niox.2011.01.007

[30] Zan L, Zhang X, Xi Y, Wu H, Song Y, et al (2014) Src regulates angiogenic factors and vascular permeability after focal cerebral ischemia-reperfusion. Neuroscience 262: 118–128. 24412374 10.1016/j.neuroscience.2013.12.060 PMC3943922

[31] Wu H, Wu T, Li M, Wang J (2012) Efficacy of the lipid-soluble iron chelator 2,2′-dipyridyl against hemorrhagic brain injury. Neurobiol Dis 45: 388–394. 21930208 10.1016/j.nbd.2011.08.028 PMC3225648

[32] Wu T, Wu H, Wang J, Wang J (2011) Expression and cellular localization of cyclooxygenases and prostaglandin E synthases in the hemorrhagic brain. J Neuroinflammation 8: 22. 21385433 10.1186/1742-2094-8-22 PMC3062590

[33] Wang J, Dore S (2008) Heme oxygenase 2 deficiency increases brain swelling and inflammation after intracerebral hemorrhage. Neuroscience 155: 1133–1141. 18674596 10.1016/j.neuroscience.2008.07.004 PMC4696610

[34] Koyama Y, Kotani M, Sawamura T, Kuribayashi M, Konishi R, et al (2013) Different actions of endothelin-1 on chemokine production in rat cultured astrocytes: reduction of CX3CL1/fractalkine and an increase in CCL2/MCP-1 and CXCL1/CINC-1. J Neuroinflammation 10: 51. 23627909 10.1186/1742-2094-10-51 PMC3675376

[35] Sparapani M, Buonamici L, Ciani E, Battelli MG, Ceccarelli G, et al (1997) Toxicity of ricin and volkensin, two ribosome-inactivating proteins, to microglia, astrocyte, and neuron cultures. Glia 20: 203–209. 9215729 10.1002/(sici)1098-1136(199707)20:3<203::aid-glia4>3.0.co;2-8

[36] Liu C, Wu J, Xu K, Cai F, Gu J, et al (2010) Neuroprotection by baicalein in ischemic brain injury involves PTEN/AKT pathway. J Neurochem 112: 1500–1512. 20050973 10.1111/j.1471-4159.2009.06561.x

[37] Wang J, Zhuang H, Dore S (2006) Heme oxygenase 2 is neuroprotective against intracerebral hemorrhage. Neurobiol Dis 22: 473–476. 16459095 10.1016/j.nbd.2005.12.009

[38] Chang CF, Cho S, Wang J (2014) (-)-Epicatechin protects hemorrhagic brain via synergistic Nrf2 pathways. Ann Clin Transl Neurol 1: 258–271. 24741667 10.1002/acn3.54 PMC3984761

[39] Vande VB, Hanssens JL (2007) Cytotoxicity of two bonding adhesives assessed by three-dimensional cell culture. Angle Orthod 77: 716–722. 17605479 10.2319/052706-212.1

[40] Barreto GE, Gonzalez J, Torres Y, Morales L (2011) Astrocytic-neuronal crosstalk: implications for neuroprotection from brain injury. Neurosci Res 71: 107–113. 21693140 10.1016/j.neures.2011.06.004

[41] Hanke ML, Kielian T (2011) Toll-like receptors in health and disease in the brain: mechanisms and therapeutic potential. Clin Sci (Lond) 121: 367–387. 21745188 10.1042/CS20110164 PMC4231819

[42] Liu H, Xin L, Chan BP, Teoh R, Tang BL, et al (2002) Interferon-beta administration confers a beneficial outcome in a rabbit model of thromboembolic cerebral ischemia. Neurosci Lett 327: 146–148. 12098656 10.1016/s0304-3940(02)00371-3

[43] Hecker M, Hartmann C, Kandulski O, Paap BK, Koczan D, et al.. (2013) Interferon-beta therapy in multiple sclerosis: the short-term and long-term effects on the patients' individual gene expression in peripheral blood. Mol Neurobiol. 10.1007/s12035-013-8463-1 23636981

[44] Bowen KK, Naylor M, Vemuganti R (2006) Prevention of inflammation is a mechanism of preconditioning-induced neuroprotection against focal cerebral ischemia. Neurochem Int 49: 127–135. 16759752 10.1016/j.neuint.2006.02.011

[45] Shi M, Deng W, Bi E, Mao K, Ji Y, et al (2008) TRIM30 alpha negatively regulates TLR-mediated NF-kappa B activation by targeting TAB2 and TAB3 for degradation. Nat Immunol 9: 369–377. 18345001 10.1038/ni1577

[46] Gesuete R, Packard AE, Vartanian KB, Conrad VK, Stevens SL, et al (2012) Poly-ICLC preconditioning protects the blood-brain barrier against ischemic injury in vitro through type I interferon signaling. J Neurochem 123 Suppl 2 75–85. 23050645 10.1111/j.1471-4159.2012.07946.x PMC3481200

[47] Pan LN, Zhu W, Li C, Xu XL, Guo LJ, et al (2012) Toll-like receptor 3 agonist Poly I:C protects against simulated cerebral ischemia in vitro and in vivo. Acta Pharmacol Sin 33: 1246–1253. 22983393 10.1038/aps.2012.122 PMC4002702

[48] Packard AE, Hedges JC, Bahjat FR, Stevens SL, Conlin MJ, et al (2012) Poly-IC preconditioning protects against cerebral and renal ischemia-reperfusion injury. J Cereb Blood Flow Metab 32: 242–247. 22086194 10.1038/jcbfm.2011.160 PMC3272611

[49] Longhi MP, Trumpfheller C, Idoyaga J, Caskey M, Matos I, et al (2009) Dendritic cells require a systemic type I interferon response to mature and induce CD4+ Th1 immunity with poly IC as adjuvant. J Exp Med 206: 1589–1602. 19564349 10.1084/jem.20090247 PMC2715098

[50] McCartney S, Vermi W, Gilfillan S, Cella M, Murphy TL, et al (2009) Distinct and complementary functions of MDA5 and TLR3 in poly(I:C)-mediated activation of mouse NK cells. J Exp Med 206: 2967–2976. 19995959 10.1084/jem.20091181 PMC2806445

